# Advances and Applications of Ion Torrent Personal Genome Machine in Cutaneous Squamous Cell Carcinoma Reveal Novel Gene Mutations

**DOI:** 10.3390/ma9060464

**Published:** 2016-06-14

**Authors:** Yu-Ping Hsiao, Chun-Te Lu, Ju Chang-Chien, Wan-Ru Chao, Jiann-Jou Yang

**Affiliations:** 1 Institute of Medicine, School of Medicine, Chung Shan Medical University, Taichung 402, Taiwan; missyuping@gmail.com (Y.-P.H.); ctlu119@vghtc.gov.tw (C.-T.L.); littleuni@hotmail.com (W.-R.C.); 2 Department of Dermatology, Chung Shan Medical University Hospital, Taichung 402, Taiwan; 3 Division of Plastic Surgery, Taichung Veterans General Hospital, Taichung 407, Taiwan; 4 Department of Biomedical Sciences, Chung Shan Medical University, Taichung 402, Taiwan; swan1204@hotmail.com; 5 Institute of Microbiology and Immunology, College of Medicine, Chung Shan Medical University, Taichung 402, Taiwan; 6 Department of Pathology, Chung Shan Medical University Hospital, Taichung 402, Taiwan; 7 Department of Medical Sciences, Chung Shan Medical University Hospital, Taichung 402, Taiwan

**Keywords:** ion torrent personal genome machine, next generation sequencing technology, squamous cell carcinoma

## Abstract

The Ion Torrent Personal Genome Machine (Ion PGM) is a semiconductor-based sequencing technology that is high quality, scalable, and economic. Its applications include genomic sequencing, drug resistance testing, microbial characterization, and targeted sequencing in cancer studies. However, little is known about the application of Ion PGM in cutaneous squamous cell carcinoma (cSCC). We therefore investigated the utility and validity of Ion PGM in cSCC and also gained a better understanding of the underlying molecular biology of cSCC. We detected novel gene mutations (KDR, FGFR2, and EGFR) in two cSCC patients. Moreover, we validated these mutations by pyrosequencing and Sanger sequencing. Our results indicated that the mutation screen using Ion PGM is consistent with traditional sequencing methods. Notably, these identified mutations were present at significantly higher rates in high-risk cSCC. Our results demonstrate a method to detect targetable genes in high-risk cSCC, and suggest that Ion PGM may enable therapeutic decision-making and future potential targets for personalized therapies in cSCC.

## 1. Introduction

Cutaneous squamous cell carcinoma (cSCC) derived from keratinocytes of the skin can be locally invasive or distantly metastatic [1,2,3]. cSCC is the leading fatal cause in non-melanoma skin cancers [1,3,4] and the second most common skin cancer, accounting for 15%–25% of all cutaneous malignancies [1,5,6]. Approximately 250,000 cSCCs are diagnosed annually in the United States and the incidence is still increasing [1,7]. Mortality from cSCC is usually due to metastasis [1,5,8] and the survival rate has not declined in decades [9]. Some hypotheses for the carcinogenesis of cSCC have been proposed [2,6,10], but the actual etiology and carcinogenesis of cSCC remains unclear [11,12].

cSCC represents a classic model of somatic mutation and a fundamentally genetic disease with highly individualized genetic etiology [13,14]. The Ion Torrent Personal Genome Machine (Ion PGM), a next generation sequencing technology, has revolutionized genetic and genomic research with the comprehensive analysis of genomes, transcriptomes and interactomes [15,16]. Based on semiconductor sequencing technology that detects electrical signals directly on a disposable chip, Ion PGM has the advantages of high accuracy, speed and affordability [17,18]. To date, little is known about the utilization of Ion PGM in cSCC. In this study, we investigated the mutational patterns of the key genes in cSCC by Ion PGM, pyrosequencing and Sanger sequencing.

## 2. Results

### 2.1. Demographic Data

We included 67 cSCC specimens from patients of ages ranging from 44 to 93 years (mean age: 73.9 years old) and of both sexes (female: 29; male: 38). Of the 67 tumors, 51 (76.1%) were from patients of advanced age, and 16 (23.9%) were from patients below 65 years old. Forty-two (62.7%) specimens came from sun-exposed sites and 25 (37.3%) came from non-sun-exposed sites. Eighteen (26.9%) of the specimens were larger than 2 cm, and six (9%) had deep invasion with Clark level IV. The ratios of histopathological grading were nine (13.4%) well differentiated, 39 (58.2%) moderately differentiated, and 19 (28.4%) poorly differentiated. There were three (4.5%) cases with distal metastasis and seven (10.4%) with recurrence. We assigned risk according to the American Joint Committee on Cancer staging manual, 7th Edition (AJCC-7), with risk features including tumor diameter 2 cm, Clark level IV, tumor thickness >2 mm, location on ear or non-hair-bearing (vermillion) lip, poorly differentiated histologic finding for the first time, and perineural invasion [1]. Thirty-seven (55.2%) were designated as high-risk cSCCs, and 30 (44.8%) were low risk (Table 1).

### 2.2. Target Variants of High-Risk cSCC Using Ion Torrent Personal Genome Machine

Among the high-risk cSCCs, two specimens were microdissected and analyzed for driver gene mutations using a panel covering 739 mutations in 46 cancer-related genes (Ion AmpliSeq Cancer Panel, Life Technologies, Carlsbad, CA, USA). In patient one, 14 different mutations were observed in 11 cancer-related genes. Among these mutations, seven were missense mutations and the rest were silent mutations (Table 2). In patient two, 14 different mutations were observed in 12 cancer-related genes. Among these mutations, seven were missense mutations and the rest were silent mutations (Table 3). Five of the seven missense mutations were found in both patients one and two: a KDR mutation (Chr. 4; g. 55972974 T > A); an EGFR mutation (Chr. 7; g. 55249110 G > A); two FGFR2 mutations (Chr. 10; g. 123274818 T > A and g. 123274819 T > C); and a HNF1A mutation (Chr. 12; g. 121432011G > C). Based on the above results, four target oncogenes (KDR, EGFR, FGFR2, and HNF1A) from the two high-risk cSCC patients were subsequently confirmed for the presence of missense gene mutations with pyrosequencing and Sanger sequencing.

### 2.3. Mutation Prevalence in cSCC According to Tumor Risk

**Figure 1:**
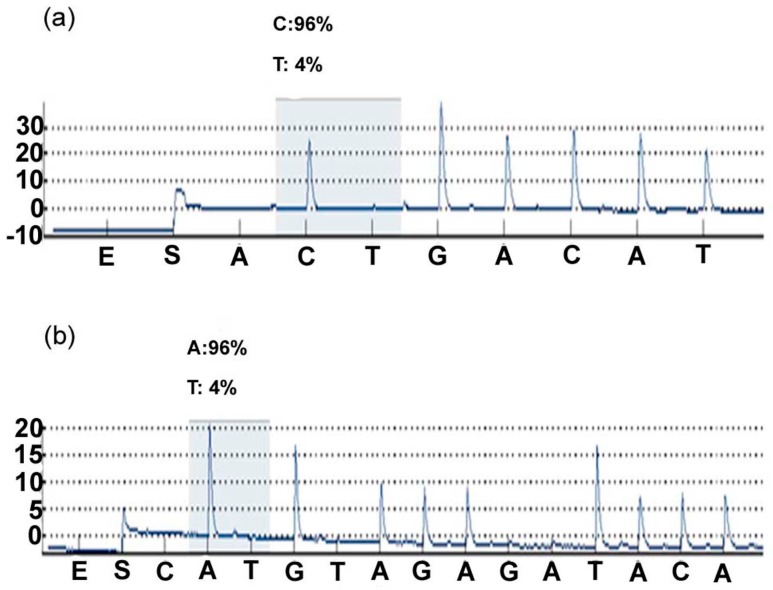
EGFR and FGFR2 mutations were validated by pyrosequencing of cSCC specimens. (**a**) EGFR g. 55249110 G > A (**b**) FGFR2 g. 123274818 T > A. Both (**a**,**b**) are reverse sequences.

**Figure 2:**
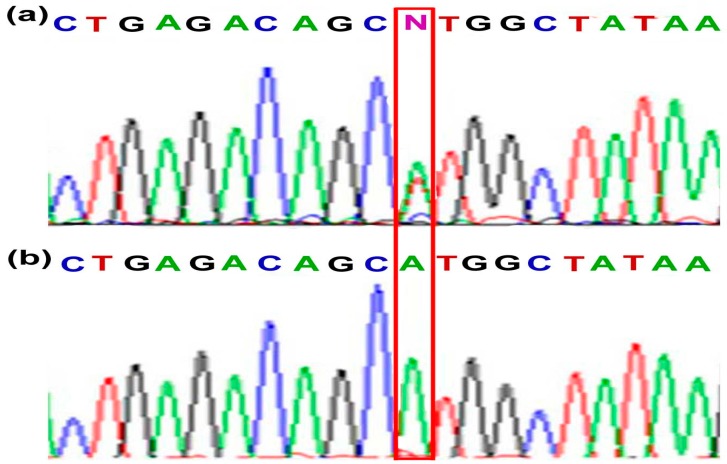
KDR mutation (g. 55972974 T > A) was validated by Sanger DNA sequencing of cSCC specimens: (**a**) heterozygous mutation (**b**) homozygous mutation.

Target genes’ missense mutations (KDR, EGFR, FGFR2 and HNF1A) were detected by PCR in 67 microdissected cSCC samples. We used pyrosequencing to detect EGFR g. 55249110 G > A and FGFR2 g. 123274818 T > A mutations in 22 (32.8%) and 28 (41.8%) of the cSCC samples (Figure 1), respectively. Simultaneously, No FGFR2 g. 123274819 T > C mutant was found in this study by pyrosequencing. In addition, the KDR g. 55972974 T > A mutant was validated by Sanger DNA sequencing (Figure 2). Our results indicated a mutation rate of 61.2% (41/67) in cSCC. Among 41 patients, 14 were homozygous (14/41; 33.3%) and 27 were heterozygous mutations (27/41; 66.7%), respectively. These results indicate that the mutation screen by Ion PGM is consistent with the traditional sequencing method. However, the FGFR2 g. 123274819 T > C mutant was not detected by pyrosequencing, nor was the HNF1A mutation detected by pyrosequencing and Sanger sequencing.

The FGFR2 mutation existed at a higher frequency in older patients with cSCC (*p* = 0.032). There were no statistically significant differences in gender, location, histopathological differentiation and metastasis for KDR, FGFR2 and EGFR mutations. Among the high-risk cSCCs, KDR, FGFR2, and EGFR mutations were detected in 72% (*p* = 0.028), 59% (*p* = 0.001), and 57% (*p* < 0.001) of high-risk cSCC, respectively (Table 4). According to the above results, the KDR g. 55972974 T > A occurred with the highest proportion. Although the FGFR2 and EGFR mutant rates are low, we consider that these mutations play some role in the tumors of older and high-risk cSCC patients. Additionally, the HNF1A mutation is most likely a sequencing artifact from the AmpliSeq Cancer HotSpot panel, as this variant is located at the end of the amplicon and is present at low frequency.

## 3. Discussion

The Ion Torrent Personal Genome Machine (Ion PGM) is a high-throughput DNA sequencing breakthrough, with applications beyond the conventional genomic sequencing which originally spurred its development [15,19,20]. This next generation sequencing technology provides rapid methods for genome-wide characterization and profiling of mRNAs, transcription factor regions, chromatin structure, metagenomics, and microbiology [15,19,21,22,23]. Ion PGM is unlike other conventional next-generation sequencing systems, which use dyes to label nucleotides and must be optically read via fluorescence imaging [17,24]. Ion PGM detects electrical signals directly on a semiconductor chip and reads nucleotides by sensing the pH value variation during complementary strand synthesis [17,24]. Whereas most sequencing techniques available nowadays take a week to process DNA samples, the Ion PGM analyzes sequences in a matter of hours [17,18]. It has surpassed traditional approaches in terms of economy, speed, and accuracy and offers comprehensive genetic information [15,19]. With shortened sequencing time, 99% accuracy [17,24], and decreased experiment costs, sequencing has become affordable for more labs and researchers, resulting in an ever-increasing array of applications [20,24].

Skin cancers involve individualized genetic etiologies with somatic alterations that underlie mosaicism [13,21,25,26]. In the past years, several genes have been considered for association with cSCCs, but genes scattered within genomes with low prevalence are difficult to detect by traditional molecular methods [13,27,28]. Deep sequencing by next generation techniques can now probe heavily mutagenized genomes with large numbers of low-frequency mutations characteristic of skin cancers [13]. To the best of our knowledge, this study is the first use of Ion PGM to reveal gene mutations in high-risk cSCCs. High-risk cSCCs are characterized by tumor size >2.0 cm, high histological grade and localization on the ear or lip, deep and perineural invasion, recurrence, immunosuppression, and arsenic toxicity, usually with additional unfavorable prognoses and poor outcome [1,4,5,29,30,31]. Scientists suggest that highly mutagenized skin cancers may stimulate additional oncogenic pathways, such that combination strategies in targeted therapies may be required [13,32,33]. Early detection and development of target therapies for high-risk cSCC are imperative. Clarifying the genetic prospect of cSCC, especially the targets which have been generally sparse, would facilitate the development of targeted therapies for cSCC [9,32].

The *KDR* gene encodes VEGFR-2, a receptor tyrosine kinase overexpressed in a variety of solid tumors [34]. *KDR* also is an important factor in tumor progression and development due to its pro-angiogenic effects [35]. Previous studies indicated that the *KDR* g. 55972974 T > A (p.Q472H) mutant had increased VEGFR-2 protein phosphorylation and was associated with increased microvessel density (MVD) in non-small cell lung cancer (NSCLC) tumor specimens [36]. *KDR* p.Q472H was identified in 7/10 lung adenocarcinomas and 1/1 mixed NSCLC/SCLC [34]. The *KDR* p.Q472H variant also appears to play a role in melanoma progression [37]. In addition, the mutation *KDR* p.Q472H also was found in Hepatic carcinosarcoma [38]. Therefore, *KDR* p.Q472H is a common variant in various tumors. Our data also implicates the *KDR* p.Q472H variant in cSCC progression. This has not been reported before and suggests that further functional studies are warranted.

## 4. Materials and Methods

### 4.1. Sample Collection

Sixty-seven Asian samples with a diagnosis of squamous cell carcinoma of the skin were obtained from the dermatological and pathologic files and tumor registry from the Department of Pathology, Chung Shan Medical University Hospital, Taiwan (2002–2012). Formalin-fixed paraffin-embedded (FFPE) tissues of cSCC were obtained from the Departments of Dermatology and Pathology, Chung Shan Medical University Hospital, Taiwan. All histological sections were reviewed and the diagnoses were confirmed by the dermatologist and pathologist. Clinical information was extracted from medical records (Table 1). The study was approved by the Chung Shan Medical University Hospital (IRB No. CS12159) institutional review board. All tumors had been previously analyzed for next-generation sequencing and status.

### 4.2. Genomic DNA Purification and Quantification

Genomic DNA was extracted from tissues after enrichment for neoplastic cellularity, using manual microdissecion, and then processed with High Pure PCR Template Preparation Kit (Indianapolis, IN, USA). Quantitation of DNA was done using a Nanodrop 1000 spectrophotometer (Thermo Scientific Nanodrop Technologies, Wilmington, NC, USA).

### 4.3. Ion Torrent Personal Genome Machine

Ten nanograms of DNA was used for multiplex PCR of a panel covering 739 mutations in 46 cancer-related genes (Ion AmpliSeq Cancer Panel, Life Technologies). Library construction of the amplicons and subsequent enrichment of the sequencing beads were performed according to the manufacturer’s protocol. While the sequencing run, the sample cSCC 1 had a coverage 2356 with 472,908 reads, and the sample cSCC 2 had a coverage 2374 with 482,928 reads. Sequencing was done on the 314 chip using the Ion Torrent Personal Genome Machine (Life Technologies) as per the manufacturer’s protocol. Data analysis, including alignment to the hg19 human reference genome and base calling, was done using built-in software v2.2.

### 4.4. Sequencing

**Figure 3:**
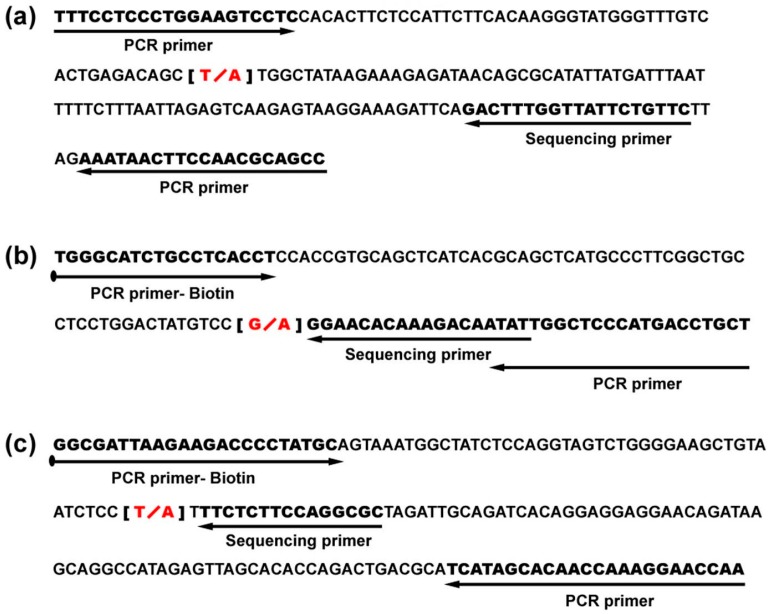
Specific primers for KDR, EGFR, and FGFR2 were used in this study: (**a**) primers for KDR mutation; (**b**) primers for mutation variant; (**c**) primers for FGFR2 mutation.

DNA extracted from the microdissected fresh-frozen skin cancers, pre-skin cancers and normal epidermis was subjected to whole genome amplification using Repli G (Qiagen, Germantown, OH, USA) and used in PCR as a template for specific primer sets (Figure 3) designed to amplify the target genes. DNA extracted from skin cancers was also amplified in PCR without undergoing prior whole genome amplification. PCR products were purified using ExoSap and used directly in Sanger sequencing with BigDye v3.1 and the Applied Biosystems 3730 sequencer for KDR and in pyrosequencing with the PyroMark sequencer for EGFR and FGFR2.

### 4.5. Statistical Analysis

All experiments were performed and presented as means ± SD. Statistical analyses were performed using one-way analysis of variance (ANOVA) followed by Tukey’s *post-hoc* test (SPSS 17.0 software) to determine significant differences among the groups. The difference was considered significant when *p* < 0.05.

## 5. Conclusions

In conclusion, we identified gene mutations in cSCC using personalized Ion Torrent technology. Furthermore, KDR, FGFR2, and EGFR gene mutations were found in high-risk cSCC, suggesting that these mutations could be potential therapeutic targets for cSCC treatment.

## Figures and Tables

**Table 1:** Demographic and histopathological data of cutaneous squamous cell carcinoma patients.

Characteristics	No. of Patients	Percentage	Characteristics	No. of Patients	Percentage
Age	-	-	Differentiation	-	-
<65	16	23.9	Poor	19	28.4
≧65	51	76.1	Moderate	39	58.2
Sex	-	-	Well	9	13.4
Male	38	56.7	Recurrence	-	-
Female	29	43.3	No	60	89.6
Location	-	-	Yes	7	10.4
Face	28	41.8	Metastasis	-	-
Ear and lip	14	20.9	No	64	95.5
Others	25	37.3	Yes	3	4.5
Tumor size	-	-	Risk	-	-
≤2 cm	49	73.1	Low	30	44.8
>2 cm	18	26.9	High	37	55.2
Clark level	-	-	-	-	-
<IV	61	91.0	-	-	-
≧IV	6	9.0	-	-	-

**Table 2:** Target variants of high-risk cSCC no. 1 specimen.

Chrom	Position	Gene Sym	Ploidy	Ref	Variant	VarFreq	Coverage	Ref Cov	Var Cov	AA Mut
chr4	1807894	FGFR3	Hom	G	A	99.64	1652	6	1646	-
chr4	55141055	PDGFRA	Hom	A	G	99.92	4967	1	4963	-
chr4	55972974	KDR	Hom	T	A	99.87	3051	3	3047	p.Q427H
chr5	112175770	APC	Hom	G	A	99.73	1472	4	1468	-
chr7	55249063	EGFR	Het	G	A	44.12	102	57	45	-
chr7	55249110	EGFR	Het	G	A	8.33	2160	1980	180	p.R803Q
chr7	116339672	MET	Het	C	T	53.44	1106	514	591	-
chr7	116340262	MET	Het	A	G	51.56	3305	1598	1704	p.N375S
chr10	43613843	RET	Het	G	T	51.06	47	23	24	-
chr10	123274818	FGFR2	Het	T	A	5.37	3797	3582	204	p.K367M
chr10	123274819	FGFR2	Het	T	C	10.88	3905	3479	425	p.K367E
chr11	108236046	ATM	Het	C	G	5.45	716	676	39	-
chr12	121432011	HNF1A	Het	G	C	4.38	3450	3292	151	p.G253A
chr14	105246407	AKT1	Het	G	A	43.66	6439	3627	2811	-

**Table 3:** Target variants of high-risk cSCC no. 2 specimen.

Chrom	Position	Gene Sym	Ploidy	Ref	Variant	Var Freq	Coverage	Ref Cov	Var Cov	AA Mut
chr3	178952190	PIK3CA	Het	C	A	7.42	1307	1209	97	-
chr4	1807894	FGFR3	Hom	G	A	99.67	1534	1	1529	-
chr4	55141055	PDGFRA	Hom	A	G	99.91	5433	4	5428	-
chr4	55152040	PDGFRA	Het	C	T	61.32	6981	2699	4281	-
chr4	55972974	KDR	Het	T	A	52.74	2300	1085	1213	p.Q427H
chr5	112175770	APC	Hom	G	A	99.24	1964	14	1949	-
chr7	55249110	EGFR	Het	G	A	6.79	3227	3004	219	p.R803Q
chr9	21971179	CDKN2A	Het	G	A	58.12	1194	499	694	p.A60V
chr10	43613843	RET	Het	G	T	43.9	41	23	18	-
chr10	123274818	FGFR2	Het	T	A	5.94	4445	4169	264	p.K367M
chr10	123274819	FGFR2	Het	T	C	11.02	4536	4027	500	p.K367E
chr11	108236046	ATM	Het	C	G	5.01	659	624	33	-
chr12	121432011	HNF1A	Het	G	C	4.52	2743	2612	124	p.G253A
chr13	48942722	RB1	Het	C	T	8.91	404	368	36	p.P370L

**Table 4:** Target genes (KDR, FGFR2, EGFR) expressions in cSCC specimens (*n* = 67).

Characteristics	No. of Patients	KDR			FGFR2			EGFR		
		Positive	Negative		Positive	Negative		Positive	Negative	
	Total	*n*	*n*	*p*-value	*n*	*n*	*p*-value	*n*	*n*	*p*-value
Age				0.200			0.032 *			0.174
<65	16	12	4		3	13		3	13	
≧65	51	29	22		25	26		19	32	
Sex				0.385			0.154			0.806
Male	38	25	13		13	25		12	26	
Female	29	16	13		15	14		10	19	
Location				0.721			0.080			0.241
Sunexposure	42	25	17		21	21		16	26	
Non-sunarea	25	16	9		7	18		6	19	
Differentiation				0.963			0.751			0.268
Poor	19	12	7		11	8		8	11	
Moderate	39	23	16		11	28		12	27	
Well	9	6	3		6	3		2	7	
Recurrence				0.820			0.096			0.001 *
No	60	37	23		23	37		16	44	
Yes	7	4	3		5	2		6	1	
Metastasis				0.845			0.379			0.985
No	64	39	25		26	38		21	43	
Yes	3	2	1		2	1		1	2	
Risk				0.028 *			0.001 *			<0.001*
Low	30	14	16		6	24		1	29	
High	37	27	10		22	15		21	16	

* The difference was considered significant when *p* < 0.05.
